# Prevalence of pain and interference with daily activities and sleep in adults with cerebral palsy

**DOI:** 10.1111/dmcn.14678

**Published:** 2020-09-19

**Authors:** Elisabet Rodby‐Bousquet, Ann Alriksson‐Schmidt, Johan Jarl

**Affiliations:** ^1^ Centre for Clinical Research Uppsala University‐Region Västmanland Västerås Sweden; ^2^ Department of Clinical Sciences Lund Orthopaedics Lund University Lund Sweden; ^3^ Department of Clinical Sciences Malmö Health Economics Lund University Lund Sweden

## Abstract

**Aim:**

To analyse the prevalence of pain, pain sites, pain severity, and pain interfering with work or daily activities and sleep in adults with cerebral palsy (CP).

**Method:**

This was a cross‐sectional study based on data from 1591 adults (16–76y, median age 25y; 879 males, 712 females; Communication Function Classification System [CFCS] levels I–V) in the Swedish Cerebral Palsy Follow‐up Program. Pain severity was rated for several body sites and pain interference with activities/work and sleep was also evaluated. Logistic regression was used to estimate the odds ratios (ORs) of the factors associated with the prevalence of pain and pain interfering with activities/work or sleep.

**Results:**

Pain was reported in 1059 of 1591 adults; a higher proportion self‐reported pain (69.9%) compared to proxy‐reported pain (62.4%). More adults classified in CFCS level I (72.5%) reported pain compared to those in CFCS levels II to V (56.5–64.9%). Adults with severe/very severe pain had a sixfold risk of pain interfering with activity/work (OR=6.68; 95% CI 4.99–8.96) and sleep (OR=6.60; 95% CI 4.84–8.98).

**Interpretation:**

Two‐thirds of adults with CP experienced pain, which is likely to be underreported in individuals who do not communicate efficiently or rely on proxy reports. Pain strongly interfered with activities and sleep; thus, it must be assessed and treated more effectively.

**Abbreviations**
CFCS: Communication Function Classification System
CPUP: Cerebral Palsy Follow‐up Program
EDACS: Eating and Drinking Ability Classification System
MACS: Manual Ability Classification System

**What this paper adds**

- Two‐thirds of adults with cerebral palsy (CP) experienced pain from a median of three body sites.
- Pain is likely to be underreported in adults who do not communicate efficiently or rely on proxy reports.
- Adults with CP in severe pain had a sixfold risk of pain interfering with activity/work and sleep.

Pain is one of the most common secondary conditions in adults with cerebral palsy (CP) with estimates of chronic pain as high as 75%.^1^ Different types of pain at various body sites are frequent in this population, with a predominance of musculoskeletal pain.^2^, ^3^ Pain has a negative influence on several factors, such as reduced mobility, lower levels of self‐care, reduced overall function, and lower health‐related quality of life.^4^, ^5^

Most of the body of work related to pain and CP to date has been performed in relation to children. Both the frequency and intensity of pain are higher in older children with CP than in younger children^6^ and pain is more often reported in females and in individuals whose gross motor function is more severely compromised.^7^ Pain sites tend to correlate with the level of gross motor function; in general, pain is most commonly reported in the lower extremities in children and adolescents with CP.^7^, ^8^ In terms of the pain experienced by adults with CP, females still seem to be disproportionally affected,^3^, ^9^ although there are exceptions.^1^ However, whether pain sites and the distribution of pain by level of gross motor function differ in adulthood is not clear. Some studies have reported that pain appears to be more evenly distributed across the level of gross motor function severity in adulthood^1^, ^3^, ^10^ than in childhood.

As in the general population, pain increases with age in individuals with CP. There are many theories as to why pain seems to increase very rapidly in this population. Several studies^9^, ^11^, ^12^, ^13^, ^14^ have shown that a decline in gross motor function over time manifested as reduced balance, walking ability, and range of motion, and an increase in physical fatigue and problems related to spasticity. In individuals with CP who walk, a crouched standing posture may lead to reduced hip and knee extensions that worsen over time due to gravity and the altered position of body segments in relation to each other.^15^ Contractures and deformities most commonly affect the spine and lower extremities and can lead to scoliosis, pelvic obliquity, hip dislocation, windswept deformity, and foot deformities.^16^, ^17^ For instance, adults with spastic CP who reported deteriorated walking function over 7 years, also reported higher pain frequencies, greater pain severity, greater effects of pain on daily activities, greater physical fatigue, and reduced balance.^11^ Moreover, individuals with CP were no less prone to other debilitating painful comorbid conditions that affect those without CP, such as arthritis, asthma, diabetes, or cardiovascular disease.^18^

The purpose of this study was to analyse the prevalence of pain, pain sites, pain severity, and pain interfering with work/other daily activities and sleep in adults with CP at all functional levels.

## METHOD

A cross‐sectional registry study was performed based on data from all adults in the Swedish Cerebral Palsy Follow‐up Program (CPUP)^19^ from 1st January 2015 to 31st December 2018.

The CPUP is a combined national follow‐up program and registry for people with CP in Sweden,^19^ which was started in southern Sweden in 1994. Since 2005, the CPUP has been a national health care program and quality registry approved by the Swedish National Board of Health and Welfare; almost all families (98%) who have a child with CP have agreed to participate.^20^ In 2009, the CPUP started offering follow‐ups to adults with CP and by 2018, 20 of 21 health care regions in Sweden had started to enrol adults and report assessment data in the registry. Most of the adults currently enrolled were not followed in the CPUP as children. The standardized follow‐ups of adults include clinical assessments of joint range of motion, scoliosis, posture, mobility, gross and fine motor functions, communication, eating and drinking abilities, spasticity, information on pain, treatments, fractures, and use of orthoses and assistive devices, as well as patient‐reported outcome measures to assess health‐related quality of life, fatigue, and fear of falling.

### Classifications and measurements

CP was defined according to Rosenbaum et al.^21^ The inclusion and exclusion criteria of CP were based on the Surveillance of Cerebral Palsy in Europe,^22^ with inclusion of all permanent but not unchanging disorders of movement and posture due to non‐progressive brain injuries before the age of 2 years. The subtypes were divided into unilateral spastic CP, bilateral spastic CP, ataxic CP, dyskinetic CP, and mixed type/unclassifiable CP.^22^ Age was calculated based on date of birth and date of examination. Age was grouped into six categories: 16 to 19; 20 to 24; 25 to 29; 30 to 39; 40 to 49; and 50 to 76 years. In general, young people aged 16 to 19 years old still attend school and live with their parents, and the twenties are formative years in terms of finding one’s position in society (e.g. higher education, family formation, job market entry) warranting a greater granularity in terms of age categories. Adults over 50 years were grouped since there were few in each age category.

Functional levels were classified by local physical therapists, occupational therapists, and speech and language pathologists who performed the examinations according to the expanded and revised version of the Gross Motor Function Classification System (GMFCS),^23^ the Manual Ability Classification System (MACS),^24^ the Communication Function Classification System (CFCS),^25^ and the Eating and Drinking Ability Classification System (EDACS).^26^ The assessment schedule for adults in the CPUP is based on the GMFCS level of the individual, where those classified in GMFCS level I are offered an examination every third year, those in GMFCS level II every second year, and adults classified in GMFCS levels III and IV every year.

Prevalence of pain (Do you experience pain*?*) was either self‐ or proxy‐reported as ‘yes’ or ‘no’. If pain was reported, pain severity was rated for the following 10 body sites: neck; back/spine; shoulder; arm/hand; hip/thigh; knee; feet/lower leg; head; stomach; or other location. No specific body diagram was used. Laterality was not accounted for and pain in the right and/or left side was calculated as one site. As applicable, pain severity (How much bodily pain have you had during the past 4 weeks?) was graded according to the Short Form Health Survey 36^27^ into one of the following response options for each relevant pain site: 1=none; 2=very mild; 3=mild; 4=moderate; 5=severe; 6=very severe. Finally, items from the Short Form Health Survey 36 were used to query those with pain as to how it had interfered with activities (During the past 4 weeks, how much did pain interfere with your normal activities and work? Including both work outside the home and housework) and sleep (During the past 4 weeks, how much did pain interfere with your sleep?) using the following response options: 1=not at all; 2=a little bit; 3=moderately; 4=quite a bit; 5=extremely.

This study was carried out in accordance with the Code of Ethics of the World Medical Association (Declaration of Helsinki) for experiments involving humans. Ethical approval was granted by the Regional Ethics Committee in Lund (no. 2009/341).

### Statistical analyses

A *χ* ^2^ test was used for univariate analysis of categorical variables. A Spearman’s rank correlation coefficient was used to analyse the relationships between the functional classifications (i.e. GMFCS, MACS, CFCS, and EDACS). Logistic regression was used to estimate the unadjusted and adjusted odds ratios (ORs) of factors associated with the prevalence of pain, pain interfering with activity/work, and pain interfering with sleep. The area under the curve and Nagelkerke pseudo *R* ^2^ were used to estimate the model fit. The prevalence of pain was dichotomized into pain or no pain. Severe pain was dichotomized into experiencing severe or very severe pain versus not experiencing severe pain. All significance tests were conducted at the 0.05 level. SPSS v26 (IBM Corp., Armonk, NY, USA) and SAS v9.4 (SAS Institute, Cary, NC, USA) were used for all statistical analyses.

## RESULTS

In total, data on 1591 adults with CP (16–76y, median age 25y; 879 males, 712 females) were reported in the database (Table 1). Information on pain was self‐reported by 1098 adults and proxy‐reported for 457; data on who reported the pain (self vs proxy) were missing for 36 adults.

**Table 1:** Characteristics, pain prevalence, and pain reported as severe or very severe by the 1591 adults with cerebral palsy (CP)

	Participants		Pain prevalence		*p*	Severe pain		*p*
	*n*	%	*n*	%		*n*	%	
Sex								
Male	879	55.2	533	60.6	<0.001	168	19.1	<0.001
Female	712	44.8	526	73.9		217	30.5	
Age group, y								
16–19	316	19.9	202	63.9	<0.029	55	17.4	<0.001
20–24	469	29.5	296	63.1		95	20.3	
25–29	291	18.3	189	64.9		69	23.7	
30–39	255	16.0	190	74.5		82	32.2	
40–49	145	9.1	102	70.3		43	29.7	
50–76	115	7.2	80	69.6		41	35.7	
CP subtype								
Spastic unilateral	346	21.8	230	66.5	0.393	87	25.1	0.614
Spastic bilateral	880	55.4	597	67.8		218	24.8	
Ataxic	74	4.7	42	56.8		17	23.0	
Dyskinetic	214	13.5	139	65.0		43	20.1	
Mixed type/unclassifiable	74	4.7	49	66.2		20	27.0	
Missing data	3							
GMCS level								
I	345	21.7	229	66.4	0.990	66	19.1	0.078
II	336	21.1	220	65.5		96	28.6	
III	241	15.1	162	67.2		59	24.5	
IV	302	19.0	203	67.2		73	24.2	
V	367	23.1	245	66.8		91	24.8	
MACS level								
I	407	25.7	278	68.3	0.407	94	23.1	0.464
II	399	25.2	272	68.2		105	26.3	
III	250	15.8	158	63.2		53	21.2	
IV	219	13.8	137	62.6		59	26.9	
V	308	19.5	208	67.5		71	23.1	
Missing data	8							
CFCS level								
I	716	46.1	519	72.5	<0.001	194	27.1	0.002
II	204	13.1	129	63.2		57	27.9	
III	186	12.0	105	56.5		40	21.5	
IV	202	13.0	131	64.9		49	24.3	
V	244	15.7	149	61.1		36	14.8	
Missing	39							
EDACS level								
I	759	55.1	515	67.9	0.734	181	23.8	0.123
II	201	14.6	138	68.7		64	31.8	
III	168	12.2	109	64.9		45	26.8	
IV	111	8.1	70	63.1		26	23.4	
V	139	10.1	97	69.8		29	20.9	
Missing data	213							
Pain report								
Self‐report	1098	70.6	767	69.9	0.004	305	27.8	<0.001
Proxy‐report	457	29.4	285	62.4		77	16.8	
Unclear or missing data	36		7	19.4		3	8.3	

GMFCS, Gross Motor Function Classification System; MACS, Manual Ability Classification System; CFCS, Communication Function Classification System; EDACS, Eating and Drinking Ability Classification System.

### Prevalence of pain and pain severity

Current pain was reported in 1059 (66.6%) adults and was more common in females (73.9%) than males (60.6%) (*p*<0.001). No significant differences in pain were found in relation to CP subtype, GMFCS, MACS, or EDACS levels (Table 1). However, significantly more adults classified in CFCS level I (72.5%) reported pain compared to those in CFCS levels II to V (56.5–64.9%) (*p*<0.001). Pain was reported by a slightly higher proportion of adults in the 30 to 39‐year‐old age range (74.5%), compared to those in the younger and older age groups (*p<*0.029). Pain was more frequently reported (*p*=0.004) by those who self‐reported (69.9%) compared to those who had someone else report as their proxy (62.4%) (Table 1).

Severe or very severe pain in the past 4 weeks was reported by 385 adults (24.2%) and was more commonly reported (*p*<0.001) by adults who self‐reported (27.8%) compared to those who relied on proxy reports (16.8%). Severe or very severe pain in the past 4 weeks was reported by a higher proportion of females, adults over 30 years of age, and by those adults classified in a higher level of communication (CFCS level I) (Table 1).

In the multivariate logistic regression analyses (Table 2), being a female was strongly associated with reporting both pain and severe pain in the past 4 weeks, whereas an association with older age was only evident for severe pain. CFCS level was strongly negatively associated with reporting both pain and severe pain, with lower communication levels being associated with less reported pain. In a model not controlling for proxy reports, which was associated with lower levels of pain and less severe pain, the association between pain and CFCS level was stronger (results not shown). The same tendency was noted for severe pain, although in this case, the proxy report explained almost all associations between CFCS level and pain. However, this could potentially be a result of the relatively few individuals in our sample reporting severe pain. GMFCS levels IV and V were associated with reporting both pain and severe pain when controlling for CFCS level and proxy report.

**Table 2:** Logistic regression of factors associated with pain prevalence and severe pain prevalence in adults with cerebral palsy (*n*=1518)

	**Pain, OR (95% CI)**	**Severe pain, OR (95% CI)**
Male (ref)	–	–
Female	1.65^a^ (1.32–2.06)	1.72^a^ (1.35–2.20)
16–19y (ref)	–	–
20–24y	0.94 (0.69–1.29)	1.18 (0.80–1.75)
25–29y	1.07 (0.76–1.53)	1.54^b^ (1.01–2.35)
30–39y	1.42^b^ (0.97–2.07)	2.06^a^ (1.35–3.13)
40–49y	1.20 (0.77–1.89)	1.66^b^ (1.01–2.73)
50–76y	1.12 (0.69–1.82)	2.40^a^ (1.44–4.01)
GMFCS level I (ref)	–	–
GMFCS level II	1.14 (0.80–1.61)	1.63^b^ (1.10–2.40)
GMFCS level III	1.36 (0.91–2.02)	1.29 (0.83–2.00)
GMFCS level IV	1.65^b^ (1.12–2.44)	1.64^b^ (1.07–2.52)
GMFCS level V	1.97^a^ (1.26–3.10)	2.98^a^ (1.79–4.97)
CFCS level I (ref)	–	–
CFCS level II	0.56^a^ (0.39–0.79)	0.88 (0.61–1.28)
CFCS level III	0.44^a^ (0.29–0.65)	0.65^c^ (0.42–1.01)
CFCS level IV	0.51^a^ (0.32–0.81)	0.86 (0.52–1.44)
CFCS level V	0.35^a^ (0.21–0.60)	0.44^b^ (0.23–0.85)
Self‐report (ref)	–	–
Proxy report	0.66^b^ (0.46–0.97)	0.49^a^ (0.31–0.77)
Constant	1.73^a^	0.15^a^

Overall correct classification of pain 63.9%; area under the curve (AUC)=0.65; Nagelkerke *R* ^2^=0.082. Severe pain 75.3%; AUC=0.67; Nagelkerke *R* ^2^=0.094.

^a^ *p*<0.01;

^b^ *p*<0.05;

^c^ *p*<0.1. OR, odds ratio; CI, confidence interval; GMFCS, Gross Motor Function Classification System; CFCS, Communication Function Classification System.

### Pain sites

Of the 1059 adults experiencing pain, 53 individuals had missing data on pain site. In most of these cases, pain information was reported by proxies who commented that it was difficult to know where the individuals experienced pain and how severe the pain was. Back pain was the most common pain site, which was reported in 528 of 1006 adults, corresponding to 33.1% of the 1591 adults in the study. Pain in the lower extremities, such as hips/thighs (*n*=451), feet/lower legs (*n*=384), or knees (*n*=362) was more frequent than pain in the upper extremities, such as shoulders (*n*=340) or arms/hands (*n*=291). Pain in the neck (*n*=335), head (*n*=316), or stomach (*n*=305) was almost as common as pain in the upper extremities. An additional 278 adults reported pain at other sites, such as teeth, ears, or pressure‐related pain.

**Figure 1:**
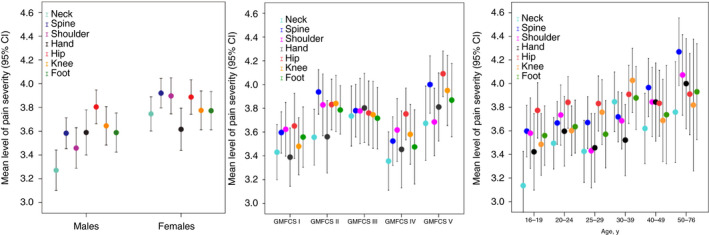
Mean level of pain severity with 95% confidence interval (CI) (2=very mild; 3=mild; 4=moderate; 5=severe; 6=very severe) for musculoskeletal pain sites: neck (*n*=335); back/spine (*n*=528); shoulder (*n*=340); arm/hand (*n*=291); hip/thigh (*n*=451); knee (*n*=362); feet/lower leg (*n*=384), based on sex, Gross Motor Function Classification System (GMFCS) level, and age.

The median number of pain sites in those in pain was three (25th to 75th centiles: 1–5), and almost three out of four adults (74.2%) reported pain in more than one site. Four or more pain sites were reported in 408 adults, of whom 319 reported four to seven pain sites and 89 reported pain in eight to 10 sites. Only 259 of the 1006 adults with reported pain sites (25.7%) reported one pain site, 191 reported two pain sites, and 148 reported three pain sites respectively. Pain severity at different pain sites is shown in Figure 1 according to sex, GMFCS level, and age. Although some trends can be noted, especially for sex, the differences were generally not statistically significant. The same tendency was seen for stomach pain, with pain severity ranging from a mean of 3.7 in males (95% CI 3.5–3.9) to 3.9 in females (95% CI 3.7 to 4.1), and from 3.7 in individuals classified in GMFCS level I (95% CI 3.4–4.0) to 4.1 in individuals classified in GMFCS level V (95% CI 3.8–4.3).

### Pain interference with daily activities/work or sleep

**Figure 2:**
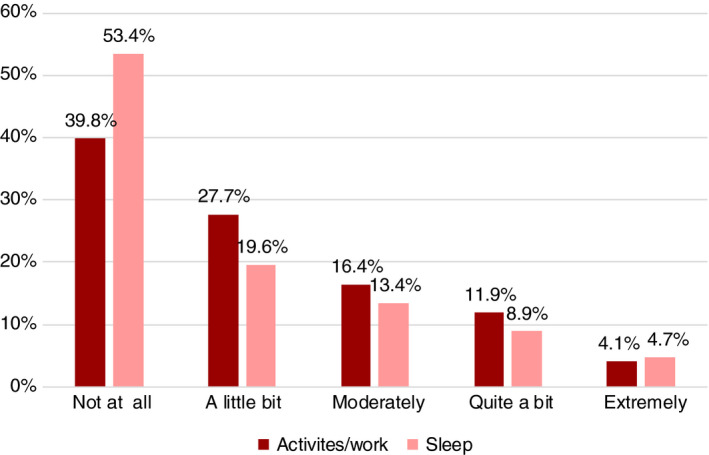
Pain interference with activities/work and sleep based on the data of 997 and 991 adults respectively of the 1059 who reported pain (missing data: *n*=62 for activities/work; *n*=68 for sleep).

Pain interfering with normal activities/work inside and outside the home was reported in 600 of 997 adults with pain (missing data, *n*=62) (Fig. 2), ranging from a little bit (*n*=276) to extremely (*n*=41). Slightly less than half of the adults, 462 of 991 (missing data, *n*=68), experienced pain interfering with sleep, ranging from a little bit (*n*=194) to extremely (*n*=47) (Fig. 2).

There was an increasing trend of more pain interfering with sleep reported in adults classified in higher GMFCS levels (*p*<0.001). Pain was reported to interfere extremely with sleep in 9.4% of the adults classified in GMFCS level V compared to 0.4% of adults classified in GMFCS level I. No other statistically significant differences were seen in pain interfering with activities/work or sleep in adults at different functional levels classified according to MACS, CFCS, EDACS, or CP subtype. However, there was a slightly higher proportion of females experiencing pain interfering with daily activities/work (*p*=0.002) and sleep (*p*<0.001) compared to males, in particular for more severe interference with daily activities/work graded as quite a bit (15.2% females vs 8.7% males) and extremely (4.5% females vs 3.8% males). The same tendency was noted for sleep, where almost twice as many females had more severe pain interfering with sleep graded as quite a bit (11.9% females vs 6.0% males) or extremely (5.7% females vs 3.8% males).

Pain interfering with daily activities/work increased with age from 29.2% in 16 to 29‐year‐olds to 48.7% in 50 to 76‐year‐olds (*p*=0.001). The same trend for age was seen for sleep (*p*=0.021), where a lower proportion of 16 to 29‐year‐olds (24.8%) reported pain interfering with sleep compared to 50 to 76‐year‐olds (34.6%). Pain severity was associated with interference with daily activities/work and sleep. Adults with severe or very severe pain had a sixfold risk of pain interfering with daily activities/work (OR=6.68; 95% CI 4.99–8.96) and sleep (OR=6.60; 95% CI 4.84–8.98). The ORs were similar after adjusting for sex and age, that is, daily activities/work (OR=6.35; 95% CI 4.73–8.53) and sleep (OR=6.38; 95% CI 4.66–8.71).

## DISCUSSION

In this study, we analysed the prevalence of pain, pain sites, pain severity, and pain interfering with activities/work and sleep in adults with CP at all functional levels. Based on our findings, pain was highly prevalent in adults with CP. In fact, two out of three adults in this study reported pain, and of those, one in four reported severe pain. The prevalence of pain in this study is similar to previous findings of pain in adults with CP in Sweden,^28^ but higher than recently reported rates for younger adults in their early twenties in Sweden (49%).^3^ This supports the notion that the frequency of pain increases rapidly during early adulthood and younger middle age in this population.

The prevalence of pain (66.5%) was slightly lower than what has been reported internationally (70–82%),^1^, ^2^, ^9^ but this may be partially explained by different inclusion and exclusion criteria, definitions of pain, and recall periods. A recent meta‐analysis^2^ of 15 studies, including a total of 1243 adults, defined pain as any current or chronic pain and showed a prevalence similar to our findings (70%). Interestingly, pain prevalence was higher in studies excluding adults with severe cognitive impairments both for current (85%)^9^ and chronic pain (75%).^1^ Pain prevalence was higher than what has been reported for children with CP,^6^ although in Sweden the same items are used to assess pain in the CPUP;^29^ however, the rates for adults are similar to international findings of pain in adolescents with CP.^30^, ^31^ Back pain was most frequently reported in the current study, which is in agreement with some previous findings.^3^, ^9^, ^11^ However, this stands in contrast to a recent systematic review,^2^ where legs were identified as the most commonly reported pain site in adults with CP. We found a higher prevalence of pain associated with the hips/thighs than what has been reported in Norway, where higher rates of pain were reported in the neck and feet.^11^ This could potentially be explained by the higher proportion of adults classified in GMFCS level V in our sample compared to the majority classified in GMFCS level I in the Norwegian study.

Of interest, pain sites appear to differ between children and adults with CP. Research on pain in children suggests that it is more frequent in the lower extremities.^6^ Pain sites also seem to differ based on GMFCS level. Pain in the feet is more common in children classified in GMFCS levels I and II than in those classified in GMFCS levels III to V, who are more likely to experience pain from the knees and hips.^6^ In adulthood, back pain seems to become more of a concern,^3^ which is also a common problem in the general population. Adults have pain in more sites than children. In adults, 75% report pain in more than one body site, whereas the corresponding numbers for children with CP vary between 36% and 66% even though the categorization of sites differs slightly between studies.^8^, ^31^ Although not assessed in this study, having pain at several sites might be indicative of a more generalized type of pain, as opposed to more local pain, which appears to be more common in children. It is also possible that over time pain is more likely to become chronic and that the increase of age‐related comorbidities and secondary conditions in this population of individuals also affects the nature of the pain experienced.

Both prevalence and severity of pain were more frequent in adults who self‐reported compared to adults with proxy‐reported pain. This differs from previous findings in adolescents with CP, where a significant agreement has been reported between self‐reported and proxy‐reported pain^31^ and speech was not associated with reporting of severe pain. In our study, significantly more adults classified in CFCS level I (72.5%) reported pain compared to individuals classified in higher CFCS levels and this association was even stronger in the multivariate analysis. Therefore, we can assume that not only the ability to communicate matters, but also that the efficiency of communication has a great impact in adults with CP. The fact that proxy reporting was associated with less pain in the multivariate analysis further emphasizes the importance of the ability to communicate effectively to accurately identify pain. This may not be very surprising, but the magnitude of the association is a cause for concern. CFCS levels II to IV are associated with half the likelihood of reporting pain (OR approximately 0.5) compared to CFCS level I and even lower for CFCS level V. It is unlikely that other unobserved factors could explain such a strong association. Therefore, the results of this study raise the question of how reliable these types of pain questions are for adults with CP. The Swedish version of the Short Form Health Survey 36 was used and it has shown good psychometric properties when evaluated in a non‐stratified, random, national sample of 18 to 75‐year‐olds; however, it has not been evaluated specifically for adults with CP in Sweden.^27^ Alternative pain measures, such as numerical rating scales, visual analogue scales, faces pain scales, body maps, or observational scales should be considered in addition to interpreting pain information stratified according to communication level. However, this may be challenging and a recent evaluation of the revised Face, Legs, Activity, Cry and Consolability scale used for adults with CP showed low agreement between self‐reported pain and pain rated by health care specialists (nurses and physicians) and between raters.^32^ A low agreement has also been reported for the Short Form Health Survey 36 where proxies tend to underestimate bodily pain in older adults with physical disabilities.^33^ There are several challenges faced by people with CP with communication limitations, particularly those reliant on augmentative and alternative communication, such as access to appropriate equipment, time to report, and health professionals’ knowledge of augmentative and alternative communication. In addition, cognitive ability, behaviour challenges, vision, hearing, and motor impairments influence the ability to communicate efficiently.

With regard to the site of pain, proxies have the additional difficulty of specifying pain site and severity. This could be even more challenging in adults where the proxy accompanying the adult to the examination could vary from family members to professional assistants. Unfortunately, we cannot distinguish between family and professional assistants. Our results contradict trends reported for children with CP, where family members appear to be highly attuned to pain in their children and overestimate rather than underestimate their pain.^31^ It may be challenging to interpret pain behaviour expressed as changes in facial or vocal expressions, eating, sleeping, personality, activity, or physiological changes. This may indicate that the difference in pain prevalence between children and adults is most likely even higher than reported. In contrast, no significant differences in pain were found regarding CP subtype, GMFCS, MACS, or EDACS levels in the univariate analyses. However, GMFCS level was associated with pain prevalence after controlling for communication ability. This means that given a certain level of communication ability, higher GMFCS level is associated with a greater likelihood of reporting both pain and severe pain, which is in contrast to some previous suggestions in the literature that pain is evenly distributed across GMFCS levels in adults with CP.^3^, ^10^ We note that failure to control for communication ability would not have identified this relationship between pain and GMFCS level.

Most of those who reported pain also reported that it interfered with daily activities/work and almost half that it interfered with sleep. This is similar to findings in children.^8^ As expected, higher pain intensity was associated with more interference. However, age also appears to be an important factor since pain interference increased with age and was considerably higher in adults who were older than 50 years old compared to individuals younger than 30 years old. Surprisingly, pain was more commonly reported in adults 30 to 39 years of age (74.5%) than in the younger and older age groups. Other factors not included or controlled for in this study, such as medications, fatigue, dysmenorrhoea, and mental health challenges may also affect the variance of age and sex.

In this study, we had access to a large population with pain information on 1591 adults with CP. Most of those individuals were not followed in the CPUP follow‐up program as children. Even though data were gathered prospectively in a systematic way, there are several limitations to this study. The main limitation is the cross‐sectional design, which cannot provide information about the causal direction. Second, the study sample includes a higher proportion of individuals classified in GMFCS level V compared to studies of total populations of children with CP. That is, adults classified in higher GMFCS levels are more likely to be included in the CPUP registry. Therefore, the sample is not representative of the general population of adults with CP. Given the positive association between GMFCS level and the prevalence of pain, the prevalence figures reported in the current study could be exaggerated and should be interpreted with caution. However, the prevalence rates reported according to subgroup and the results regarding the factors associated with pain and pain interfering with daily activities/work and sleep are expected to be unbiased.

## Conclusions

Pain is widespread in adults with CP in Sweden across all ages, subtypes, and functional levels. Our results highlight the importance of pain assessment in adults and challenge us to assess pain more carefully in individuals who do not communicate efficiently or rely on proxy reports of pain and pain severity. Given that pain strongly interferes with life at all hours of the day, it is important, despite the difficulties, to assess and try to mitigate pain in adults with CP.

## Data Availability

Data available on request due to ethical restrictions The data that support the findings of this study are available on request from the corresponding author. The data are not publicly available due to ethical restrictions of the CPUP registry.
